# Commentary: Adverse event profiles of PARP inhibitors: analysis of spontaneous reports submitted to FAERS

**DOI:** 10.3389/fphar.2023.1241524

**Published:** 2023-08-16

**Authors:** Jeanne M. Schilder, Amanda Golembesky, Tirza Areli Calderón Boyle, Gui Lan Ye, Judi Kuplast

**Affiliations:** ^1^ GSK, Philadelphia, PA, United States; ^2^ GSK, Durham, NC, United States; ^3^ GSK, Collegeville, PA, United States

**Keywords:** niraparib, PARP inhibitors, pharmacovigilance, FAERS, lymphangioleiomyomatosis

## Introduction

Poly(ADP-ribose) polymerase inhibitors (PARPis) are effective treatments in cancers associated with underlying homologous recombination deficiency. Understanding and accurately characterizing their safety profiles is essential to provide comprehensive information for optimal patient care.

Tian et al. endeavored to characterize the safety profiles of four PARPis (niraparib, olaparib, rucaparib, and talazoparib) in a disproportionality analysis using the US FDA’s Adverse Event Reporting System (FAERS) database (Tian et al., 2022). They identified 24,141 FAERS reports listing PARPis as a primary/secondary suspect and calculated reporting odds ratios (RORs) for multiple adverse events (AEs) based on Medical Dictionary for Regulatory Activities (MedDRA) preferred terminology. Duplicate reports were removed based on identification number, and cases/non-cases were represented by AEs mentioning PARPis as suspected *versus* all other AEs.

As part of our standard review to monitor the safety of our marketed product (niraparib [Zejula]), GSK noted the high ROR between lymphangioleiomyomatosis (LAM) and niraparib (ROR = 471.20) reported in the article by Tian et al. Extreme RORs can be misleading and merit further scrutiny because of the potential volatility of disproportionality scores (A. Bate, PhD, GSK, written communication, 26 April 2023). As such, the high ROR for LAM warranted further investigation, and several limitations should be considered to appropriately contextualize the data. Notably, our investigation identified inadequacies in deduplication efforts, leading to erroneous results.

## Limitations of spontaneous AE reporting

FAERS is a large, publicly available database of spontaneous AE reports, medication error reports, and product quality complaints designed to support postmarketing safety surveillance (US FDA, 2018; Guo et al., 2022; Khaleel et al., 2022). Reports are based on suspected associations and may name multiple medications (Almenoff et al., 2005). FAERS and other spontaneous-reporting systems have well-known limitations, including incomplete data, report duplication, lack of a denominator to estimate population-based incidence, and lack of a proven, causal relationship between drugs and reported events; therefore, findings from studies leveraging FAERS data may be subject to multiple biases (Almenoff et al., 2005; Sakaeda et al., 2013; US FDA, 2018; Khaleel et al., 2022). Nevertheless, FAERS is a publicly available, well-accepted and essential safety surveillance tool, and implementation of best practices around data integrity, research methodology, and transparency are critical for accuracy and credibility.

## Case study: duplicate reports

FAERS collects reports from healthcare professionals, consumers, and manufacturers (US FDA, 2018). Individuals who have observed, heard about, or suspect they have experienced an adverse drug reaction may provide spontaneous AE reports, and multiple sources may report the same incident (Almenoff et al., 2005). Thus, duplicate reports are a significant limitation of FAERS (Hauben et al., 2007; US FDA, 2018), and thorough deduplication is a prerequisite for all analyses (Khaleel et al., 2022). As deduplication is often complicated by incomplete event records, detailed interrogation is recommended, including visual comparison of data (Hauben et al., 2007; Hauben et al., 2021). Duplicate reports compromise signal detection in disproportionality analyses and may result in an erroneously large signal of disproportionality (Hauben et al., 2021). Tian et al. report excluding duplicates based on identification number. When approached for clarification, the authors responded but declined to share additional information on their findings and deduplication processes. Unfortunately, exclusion based on identification number alone would be insufficient, as demonstrated below.

Tian et al. identified 16 reports of LAM associated with niraparib from the FAERS dataset (December 2014–October 2021), leading to an ROR of 471.20. No cases identified were associated with the other PARPis. LAM is a rare, progressive, systemic disease characterized by cystic lung destruction with a median prevalence of 4.9 per million women across seven countries (Harknett et al., 2011). Because LAM has not previously been associated with PARPis, the finding warranted further investigation.

Compared with the 16 reports identified by Tian et al., our search of the FAERS Public Dashboard identified 14 reports (accessed via the FDA Adverse Event Reporting System [FAERS] Public Dashboard). Following Commonwealth Vigilance Workbench system automated deduplication (CVW Data Mining Build 6.0.2.60) of the FAERS dataset through 1 April 2022, only six reports were identified. Commonwealth uses a quantitative method to identify pairs of duplicate case reports. This method is based on the “hit-miss” statistical algorithm described by Norén et al. in their article about duplicate detection (Norén et al., 2007). To investigate further, we obtained case information for the six FAERS reports through a Freedom of Information Act request (FDA FOIA Request Form). Available data from all six FAERS cases for event date, age, body weight, country, niraparib start/end dates, niraparib lot number, and suspected and concomitant medications were identical, strongly suggesting that all six FAERS cases were duplicates of a single case (Table 1). We also searched the GSK Global Safety Database through 22 July 2022 for reports of patients receiving niraparib that contained the MedDRA preferred term of LAM and found one report. The available FAERS case data match details of the single LAM case reported in the GSK database.

**TABLE 1:** Potential case duplication: identical data in the six cases of lymphangioleiomyomatosis reported in FAERS^a^.

Case report^b^		Country	Age and body weight	Event date	Niraparib start date and end date	Medical history	Niraparib lot number	Suspect drugs	
1	14869582^c^	Data provided	Data provided	Data provided	Data provided	Data provided	1705067	• Niraparib	• Doxorubicin
								• Carboplatin	• Trabectedin
								• Cisplatin	• Gemcitabine
								• Paclitaxel	
2	14915366	Same country as case 1	Same age and body weight as case 1	Same event date as case 1	Same start and end dates as case 1	Same medical history as case 1	1705067	• Niraparib	• Doxorubicin
								• Carboplatin	• Trabectedin
								• Cisplatin	• Gemcitabine
								• Paclitaxel	
3	14987775	Same country as case 1	Data not reported	Data not reported	Same start and end dates as case 1	Same medical history as case 1	1705067	• Niraparib	• Doxorubicin
								• Carboplatin	• Trabectedin
								• Cisplatin	• Gemcitabine
								• Paclitaxel	
4	15017724	Same country as case 1	Data not reported	Data not reported	Same start and end dates as case 1	Same medical history as case 1	1705067	• Niraparib	• Doxorubicin
								• Carboplatin	• Trabectedin
								• Cisplatin	• Gemcitabine
								• Paclitaxel	
5	15192012	Same country as case 1	Same age and body weight as case 1	Same event date as case 1	Same start and end dates as case 1	Same medical history as case 1	1705067	• Niraparib	• Doxorubicin
								• Carboplatin	• Trabectedin
								• Cisplatin	• Gemcitabine
								• Paclitaxel	
6	18429944	Same country as case 1	Data not reported	Data not reported	Same start and end dates as case 1	Same medical history as case 1	1705067	• Niraparib	• Doxorubicin
								• Carboplatin	• Trabectedin
								• Cisplatin	• Gemcitabine
								• Paclitaxel	

^a^ Personally identifiable information masked for patient privacy.

^b^ One case reported by Tesaro (now GSK); five cases reported by other manufacturers. Reports from other manufactures are tied to suspected and concomitant medications in this case.

^c^ Case reported to FAERS from GSK database.

We concluded that the ROR calculation from Tian et al. is likely erroneous because the presence of duplicate cases inflated the numerator to 16 instead of 1. With an observed count of 1, disproportionality scores are notoriously volatile, and use of Bayesian statistics are recommended to protect from oversensitivity (DuMouchel, 1999; Bate and Evans, 2009). This example of how duplicate reports can affect disproportionality analyses highlights the importance of medical review and clinical judgment when interpreting FAERS-based analyses. Additional review is particularly relevant with rare events for which deduplication is possible. Such results are best viewed as hypothesis generating (Almenoff et al., 2005; Bate and Evans, 2009).

## Additional considerations

Tian et al. concluded with a comparison of PARPi AE profiles; however, comparing products based on spontaneous AE data is not recommended (Almenoff et al., 2005; Bate and Evans, 2009). Beyond methodological challenges, variability in the dataset further limits product comparisons. Tian et al. used FAERS data from October 2014 through December 2021. While this period captures entry of multiple PARPis into the postmarketing setting, the length of commercial availability and, thus, the number of patients and duration of treatment, varied substantially between drugs. Reporting practices may also change over time (Bate and Evans, 2009).

## Conclusion

Understanding the safety profile of niraparib and other PARPis is crucial for informed decision-making. While we acknowledge the contribution of Tian et al., it is critically important to deduplicate with rigor and interpret findings with caution, considering the limitations of the FAERS database and the methodological approaches. FAERS pharmacovigilance studies can offer important insights into the safety profiles of marketed medicinal products, and conducting these analyses with best practices and transparency is crucial for the generation of rigorous findings that meaningfully impact patients’ lives.
